# How parasitic larvae affect the brain

**DOI:** 10.7554/eLife.91149

**Published:** 2023-08-23

**Authors:** Zin-Juan Klaft, Chris Dulla

**Affiliations:** 1 Department of Neuroscience, Tufts University School of Medicine Boston United States

**Keywords:** parasitic infection, neurocysticercosis, epilepsy, glutamate signaling, Taenia solium, Human, Mouse, Other

## Abstract

The release of the neurotransmitter glutamate by the parasitic tapeworm *Taenia solium* appears to be implicated in the pathophysiology of a widespread, but neglected, form of adult-onset epilepsy.

**Related research article** de Lange A, Tomes H, Selfe J, Prodjinotho UF, Verhoog MB, Mahanty S, Smith K, Horsnell W, Sikasunge C, Prazeres da Costa C, Raimondo JV. 2023. Cestode larvae excite host neuronal circuits via glutamatergic signaling. *eLife* **12**:RP88174. doi: 10.7554/eLife.88174.

Infections caused by the tapeworm *Taenia solium* are a major source of illness, especially in low- and middle-income countries (Torgerson et al., 2015). When the larvae of this parasite establish cysts in the brain, a condition that is known as neurocysticercosis, the consequences can include seizures and epilepsy. Indeed, neurocysticercosis is among the leading causes of adult-onset epilepsy worldwide (Ta and Blond, 2022; Nash et al., 2013), and a significant fraction (22–29%) of all epilepsy patients in sub-Saharan countries have neurocysticercosis (Owolabi et al., 2020; Ndimubanzi et al., 2010).

Seizures and epilepsy are thought to occur when the cysts burst and release their contents into the brain, and previous research has focused on the role of the brain’s neuroinflammatory response in the development of these conditions (Robinson et al., 2012). Interestingly *Taenia solium* larvae are thought to actively suppress the local immune response to their own presence, allowing them to reside in the brain for months or even years. However, besides the work on neuroinflammation, there has been little research into the effects of the larvae (and their secretions) on neuronal activity. Now, in eLife , Joseph Raimondo (University of Cape Town) and colleagues – including Anja de Lange and Hayley Tomes as joint first authors – report the results of experiments that explore the impact of larvae on the brain (de Lange et al., 2023). These results are directly relevant to understanding the pathogenesis of acute seizures (ictogenesis) and – since seizures beget seizures – also chronic epilepsy.

The key finding of the new work is that the larvae and their secretions contain a neurotransmitter called glutamate, with the level of glutamate being high enough to directly activate surrounding neurons. To show this, de Lange et al. first homogenized *Taenia solium* larvae and collected their excretion/secretion products. They then exposed neurons to these products, and showed that this exposure activated the neurons to fire action potentials. Moreover, if glutamate receptors in the neurons were blocked before exposure to the larval products, the neurons were not activated.

The researchers – who are based in Cape Town and at institutions in Australia, France, Germany, the UK and Zambia – then used fluorescent calcium imaging to study how local activation of neurons by the glutamate from the larvae affects local brain circuits. Rises in intracellular calcium are a proxy for neuronal activity, so imaging calcium allows the neuronal activity across brain circuits to be visualized. These imaging experiments confirmed that glutamate from the larvae caused local neuronal activation that led to the subsequent activation of synaptically connected neurons across distal brain circuits. The researchers also investigated other products excreted or secreted by the larvae that could potentially affect the firing of neurons (such as substance P, acetylcholine and potassium), but none of these had the same widespread impact as glutamate. The results were consistent, therefore, with glutamate from the larvae having a potential role in the generation and/or propagation of seizures.

Next, de Lange et al. showed that the larval products showed similar excitatory effects in in vitro brain tissue from both animal models and from resected human brain tissue. They also demonstrated that the glutamate can excite the surrounding brain tissue, which has been shown to drive later epilepsy in other studies (Zeidler et al., 2018). Moreover, it has previously been shown that glutamate released by brain tumors can induce epileptic activity (Buckingham et al., 2011; Sontheimer, 2008), and researchers are exploring ways to target glutamate release in order to prevent such seizures (Ghoochani et al., 2016).

Similar strategies may be beneficial when tackling neurocysticercosis to reduce seizures, or possibly even prevent the development of chronic epilepsy. This is critically important because millions of neurocysticercosis patients suffer from seizures and epilepsy. However, the latest study does not demonstrate that larval products cause seizures or epilepsy, as it was carried out at the level of single cells and small circuits with small volumes (just picoliters) of larval products, so for now we only know how neurocysticercosis leads to neuronal hyperexcitability. Future studies will be needed to understand the effects of larval products, and the larvae themselves, on larger circuits in vivo, and to explore if/how this pathological excitation leads to long-term changes in the brain that cause it to generate spontaneous recurring seizures. Non-glutamatergic mechanisms, including neuroimmunological processes, may also still be relevant to these changes.

This study moves the field closer to understanding epilepsy in human neurocysticercosis by providing exciting experimental evidence, and by also providing naturalistic, disease-relevant models that will enable the study of novel treatment approaches. It is also important because neurocysticercosis – a condition that disproportionately affects people in low-income and under-resourced countries – has not received sufficient attention in the past. The current findings from Cape Town may just have kickstarted much-needed research in neurocysticercosis by finding out what *Taenia solium* larvae do to the neuronal networks that surround them. Exciting!

**Figure 1.**
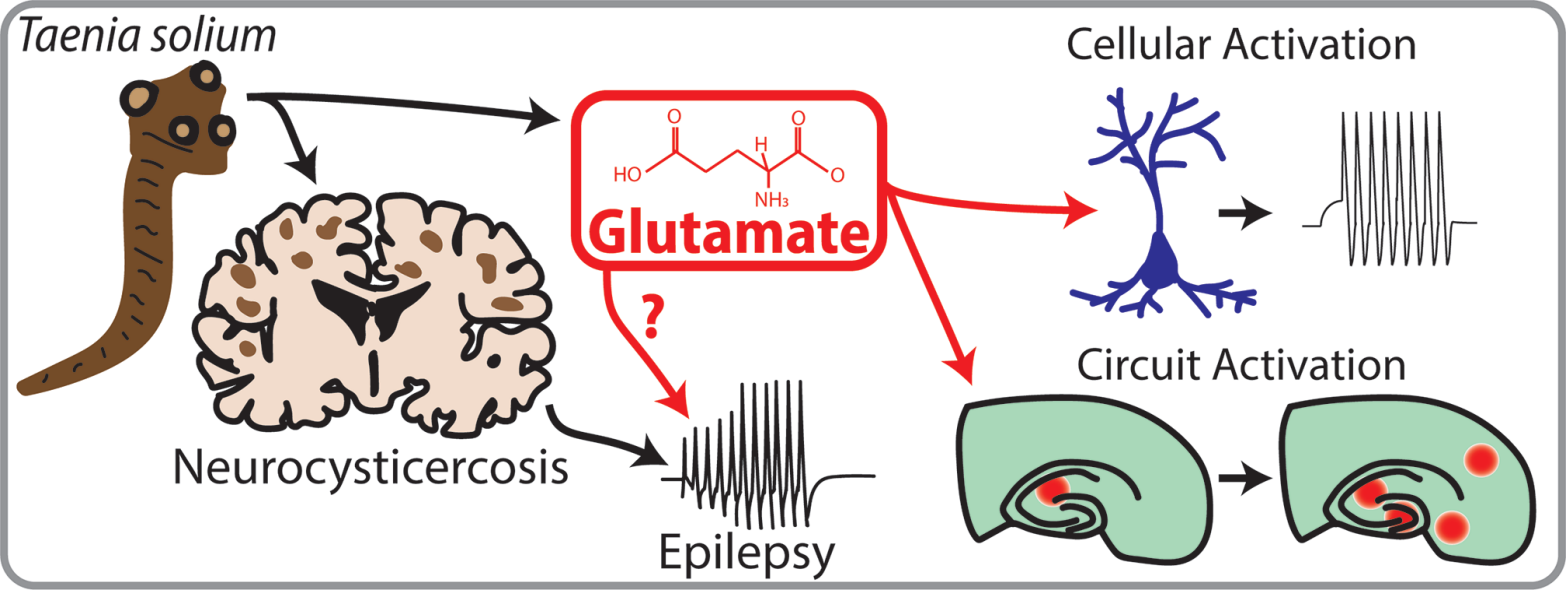
Neurocysticercosis and epilepsy. *Taenia Solium* is a parasite that can infect the brain and cause neurocysticercosis, which is a prevalent but poorly understood cause of acquired epilepsy. de Lange et al. have shown that *Taenia Solium* contains significant amounts of a neurotransmitter called glutamate, which can activate neurons and circuits of neurons. Future studies are required to establish a link between *Taenia Solium*, glutamate and epilepsy, but this study is an important first step in this direction.
